# Ulcerative Colitis, LAIR1 and TOX2 Expression, and Colorectal Cancer Deep Learning Image Classification Using Convolutional Neural Networks

**DOI:** 10.3390/cancers16244230

**Published:** 2024-12-19

**Authors:** Joaquim Carreras, Giovanna Roncador, Rifat Hamoudi

**Affiliations:** 1 Department of Pathology, School of Medicine, Tokai University, 143 Shimokasuya, Isehara 259-1193, Japan; 2 Monoclonal Antibodies Unit, Spanish National Cancer Research Center (CNIO), Melchor Fernandez Almagro 3, 28029 Madrid, Spain; groncador@cnio.es; 3 Department of Clinical Sciences, College of Medicine, University of Sharjah, Sharjah P.O. Box 27272, United Arab Emirates; rhamoudi@sharjah.ac.ae; 4 Biomedically Informed Artificial Intelligence Laboratory (BIMAI-Lab), University of Sharjah, Sharjah P.O. Box 27272, United Arab Emirates; 5 Center of Excellence for Precision Medicine, University of Sharjah, Sharjah P.O. Box 27272, United Arab Emirates; 6 Division of Surgery and Interventional Science, University College London, London NW3 2PF, UK; 7 ASPIRE Precision Medicine Research Institute Abu Dhabi, University of Sharjah, Sharjah P.O. Box 27272, United Arab Emirates

**Keywords:** artificial intelligence, machine learning, neural network, computer vision, deep learning, pre-trained model, ImageNet, ResNet-18 network, ulcerative colitis, adenocarcinoma

## Abstract

Background: Ulcerative colitis is a chronic inflammatory bowel disease of the colon mucosa associated with a higher risk of colorectal cancer. Objective: This study classified hematoxylin and eosin (H&E) histological images of ulcerative colitis, normal colon, and colorectal cancer using artificial intelligence (deep learning). Methods: A convolutional neural network (CNN) was designed and trained to classify the three types of diagnosis, including 35 cases of ulcerative colitis (n = 9281 patches), 21 colon control (n = 12,246), and 18 colorectal cancer (n = 63,725). The data were partitioned into training (70%) and validation sets (10%) for training the network, and a test set (20%) to test the performance on the new data. The CNNs included transfer learning from ResNet-18, and a comparison with other CNN models was performed. Explainable artificial intelligence for computer vision was used with the Grad-CAM technique, and additional LAIR1 and TOX2 immunohistochemistry was performed in ulcerative colitis to analyze the immune microenvironment. Results: Conventional clinicopathological analysis showed that steroid-requiring ulcerative colitis was characterized by higher endoscopic Baron and histologic Geboes scores and LAIR1 expression in the lamina propria, but lower TOX2 expression in isolated lymphoid follicles (all *p* values < 0.05) compared to mesalazine-responsive ulcerative colitis. The CNN classification accuracy was 99.1% for ulcerative colitis, 99.8% for colorectal cancer, and 99.1% for colon control. The Grad-CAM heatmap confirmed which regions of the images were the most important. The CNNs also differentiated between steroid-requiring and mesalazine-responsive ulcerative colitis based on H&E, LAIR1, and TOX2 staining. Additional classification of 10 new cases of colorectal cancer (adenocarcinoma) were correctly classified. Conclusions: CNNs are especially suited for image classification in conditions such as ulcerative colitis and colorectal cancer; LAIR1 and TOX2 are relevant immuno-oncology markers in ulcerative colitis.

## Simple Summary

Inflammatory bowel disease includes ulcerative colitis and Crohn’s disease. Ulcerative colitis affects the colon; its pathogenesis involves genetic susceptibility, microbes, and immune dysregulation, and a higher risk of colorectal cancer. This study classified images of ulcerative colitis using deep learning. A dataset was created to process images of the large intestine capturing the three diagnoses of ulcerative colitis, colorectal cancer (adenocarcinoma), and normal colon. The convolutional neural network (CNN) was trained to classify the images into three diagnostic classes, and the performance was tested on an independent dataset. The gradient-weighted class activation mapping (Grad-CAM) heatmap technique was used to understand the classification decisions. Finally, LAIR1 and TOX2 expressions were analyzed in the ulcerative colitis cases. In conclusion, the network classified the three diagnoses with high performance, and LAIR1 and TOX2 were found to correlate with the severity of ulcerative colitis.

## 1. Introduction

The aim of this study was to classify hematoxylin and eosin (H&E) images of ulcerative colitis, colorectal cancer (adenocarcinoma), and normal colon using a convolutional neural network (CNN) and the transfer learning strategy. The CNN that was used was the pre-trained ResNet-18, but other CNN models were also tested for comparison. The analysis also included immunohistochemical images of two immuno-oncology markers, LAIR1 and TOX2. In the case of ulcerative colitis, the distinction between steroid-requiring and mesalizine-responsive types was also analyzed. The study also performed conventional histopathological analysis of the samples and markers to strengthen the clinical implications of the study.

Inflammatory bowel disease is a chronic inflammatory condition characterized by relapsing-remitting inflammation of the gastrointestinal tract. It includes two main entities: ulcerative colitis, which affects the colon, and Crohn’s disease, which can involve any part of the gastrointestinal tract [1]. The estimated prevalence of inflammatory bowel disease is up to 0.8% in countries such as the UK [2]. The pathogenesis of this condition is still not well understood in either entity, and there is overlap between the two [3]. In pathogenesis, both the host immune response and microbial factors are involved, including alterations of the epithelial barrier [4], dysregulation of immune cells, dysregulation of secreted mediators, microbes, and genetic susceptibility [5] (Table 1).

Ulcerative colitis is an inflammatory disease limited to the colon that usually affects the rectum and extends to the proximal side. The prevalence of ulcerative colitis is estimated to be 5 million cases worldwide [45]. The onset of the disease is usually gradual and progressive over time and is typically accompanied by diarrhea. Systemic symptoms include weight loss, fatigue, and fever [5,45]. The disease severity ranges from mild to moderate and severe, and the Mayo scoring system/Disease Activity Index (DAI) can be used to assess disease severity and monitor the response to therapy [46,47]. The variables of the Mayo score are stool pattern, most severe rectal bleeding of the day, endoscopic findings, and global assessment by the clinician [48].

Acute complications of ulcerative colitis include severe bleeding that occurs in up to 10% of patients, fulminant colitis and toxic megacolon, and perforation [5,45]. Ulcerative colitis is a primary disease of the colon. However, extraintestinal manifestations are also present: musculoskeletal (arthritis and arthropathy), eye (uveitis and episcleritis), skin (erythema nodosum and pyoderma gangrenosum), hepatobiliary (primary sclerosing cholangitis, fatty liver and autoimmune liver disease), hematopoietic/coagulation (thromboembolism), and pulmonary [5,45,49,50,51,52,53,54].

The diagnosis of ulcerative colitis is based on the presence of chronic diarrhea for >4 weeks and evidence of colon inflammation on histological biopsy [45]. Because these characteristics are not specific, other diseases must be excluded before the diagnosis of ulcerative colitis, including Crohn’s disease, infection colitis, radiation colitis, diverticulitis, diversion colitis, solitary rectal ulcer syndrome, graft-versus-host disease, and medication-associated colitis [5]. Of note, patients with ulcerative colitis have a higher risk of developing dysplasia and colorectal cancer [5]. The extent of the affected area (patients with pancolitis) and the duration of the disease are the two major risk factors associated with the development of neoplasia [55].

The aim of treatment in patients with active ulcerative colitis is to achieve clinical and endoscopic remission. Mesalamine (mesalazine) is a 5-aminosalicylic acid derivative initially used. If there is no improvement, glucocorticoids are used (for example, budesonide or prednisone). Failure to respond and refractory disease may require systemic glucocorticoids and biological agents, such as anti-tumor necrosis (TNF) agents, anti-α4β7-integrin (vedolizumab), anti-interleukin antibody-based therapy, sphingosine-1-phosphate (S1P) receptor modulators, or small molecules (tofacitinib, a small-molecule Janus kinase inhibitor) [56,57,58,59,60,61,62,63,64,65,66].

Colorectal cancer (CRC) is a common and lethal disease, with an annual incidence of approximately 153,000 cases [67]. The risk of CRC depends on environmental and genetic factors such as inflammatory bowel disease [68]. The diagnosis is usually made by colonoscopy. The management of localized disease involves surgical resection and adjuvant chemotherapy [69].

Convolutional neural networks for deep learning image classification are an application of digital pathology and artificial intelligence in translational medicine and clinical practice [70]. The deep learning workflow includes data preprocessing, network building, training, network performance improvement by tuning hyperparameters or running multiple trials, and visualization and verification of network behavior during and after training [71].

This study used several convolutional neural networks to classify images of ulcerative colitis and differentiate between colonic control and colorectal cancer (adenocarcinoma). In addition, the protein expression of a new immuno-oncology marker was explored in ulcerative colitis cases.

## 2. Materials and Methods

### 2.1. Patients and Samples

The hematoxylin and eosin (H&E) histological slides of 35 ulcerative colitis patients were retrieved from our previous publication [72]. All patients were started on 5-ASA (mesalazine) treatment with or without probiotics. A more aggressive treatment (prednisolone as the first choice) was used if the initial treatment failed to induce remission state (UC-DAI score, 1–2) or if the disease relapsed as defined by symptoms (UC-DAI  >  2, with bloody stool) as well as by laboratory data and/or colonoscopy [72]. After a follow-up of 2 years, 35 cases were classified into two groups: 22 cases of mesalazine-responsive ulcerative colitis (clinically denominated as “benign”) and 13 cases of steroid-requiring ulcerative colitis (clinically characterized by a more “aggressive” behavior).

Other recorded clinicopathological characteristics included age at biopsy, sex, biopsy location, Baron score for endoscopic grading of ulcerative colitis [73,74,75], and histopathological Geboes score for ulcerative colitis [76]. This study was conducted in accordance with the World Medical Association (WMA) Declaration of Helsinki on ethical principles for medical research involving human subjects (IRB 13R-119, IRB14R-080, and IRB20-156).

### 2.2. Immunohistochemistry

Immunohistochemistry targeting LAIR1 was performed using a Bond-Max fully automated immunohistochemistry and in situ hybridization staining system, following the manufacturer’s instructions (Leica Biosystems K.K., Tokyo, Japan). For immunohistochemistry using DAB chromogen and hematoxylin counterstain, a polymer detection system DS9800 was used (Leica Byosystems). The primary antibody, targeting the leukocyte-associated immunoglobulin-like receptor (LAIR1/CD305), was developed by Dr. Giovanna Roncador of the Monoclonal Antibodies Core Unit, located at the Spanish National Cancer Research Center (also known as CNIO: Centro Nacional de Investigaciones Oncologicas). LAIR1 is a rat monoclonal antibody (clone JAVI82A) antigen using RBL-1-LAIR1-MYC-DDK-transfected cells, with the final booster containing LAIR1 recombinant protein (Gln22-His163, with a C-terminal 6-His tag); isotype IgG2a; reactivity, human; localization, membrane.

The TOX2 antibody targeted the TOX High Mobility Group Box Family Member 2 and was also developed by CNIO. Properties: clone name TOM924D, rat monoclonal, IgG2b K, antigen HIS-SUMO-hTOX2-Strep-tag2 full-length protein, human reactivity, nucleus localization. Conventional immunohistochemical analysis was performed using digital image quantification with Fiji software (Release 2.16.0), as we have previously described [77,78].

### 2.3. Image Classification Using CNN

This study classified images using CNNs [79]. The slides were scanned using a NanoZoomer S360 virtual slide scanner (#C13220-01, Hamamatsu Photonics, Hamamatsu, Japan). Digital whole-slide images were visualized using the NDP.view2 software (#U12388-01, Hamamatsu Photonics), and each intestinal biopsy was exported into a jpeg file at 200× magnification and 150 dpi. Subsequently, the whole-slide images were split at 243 × 243 size (PhotoScape v3.7, website: http://www.photoscape.org, last accessed on 4 December 2024), reviewed by the pathologist (J.C.), and unproductive image patches were excluded from the analysis.

The filtering criteria were as follows: (1) image patches that were not 243 × 243 pixels in size; (2) image patches smaller than 5–31 KB, which usually do not contain tissue; (3) image patches that did not contain diagnostic areas based on histopathological criteria; (4) image patches with artifacts, such as broken or folded tissue, or incorrect staining. The dataset included 18 cases of colorectal cancer (adenocarcinoma) and 21 patients classified as colon controls, all of whom had undergone diagnostic biopsies and surgical resection. For each diagnosis, all image patches from all cases were initially pooled into a single folder.

A CNN was designed based on transfer learning from ResNet-18 and trained to classify three diagnostic classes: ulcerative colitis (n = 9281), colon control (n = 12,246), and colorectal cancer (n = 63,725). The data were partitioned into a training set (70% of the image patches) to train the network, a validation set (10%) to test the performance of the network during training, and a test set (20%) as a holdout (new data) to test the performance on new data. The order of the image patches was randomized to ensure that learning across classes was even.

Data normalization was applied to the input images as previously described [71]. The code was run in the MATLAB programming language and the numeric computing environment (R2023b, update 9 released 30 July 2024, MathWorks, Natick, Apple Hill Campus, 1 Apple Hill Drive, Natick, MA 01760-2098, USA), as we have recently described [71]. The input size was 224-by-224 (224 × 224 × 3). In summary, the code loaded the pre-trained CNN, replaced the final layers, trained the network, made predictions, assessed the accuracy, and deployed the results. The design and training parameters of the CNN are listed in Table 2.

Advanced explainable artificial intelligence for computer vision was performed to understand the classification decisions by the deep learning network using the gradient-weighted class activation mapping (Grad-CAM) heatmap technique. The performance was calculated using the confusion matrices as we have previously described [80].

Using transfer learning, the performance of the ResNet-18-based network was compared to other pre-trained networks, including AlexNet, DenseNet-201, EfficientNet-b0, GoogLeNet, Inception-v3, MobileNet-v2, NASNet-Large, NASNet-Mobile, ResNet-18, ResNet-50, ResNet-101, ShuffleNet, VGG-16, VGG-19, and Xception (Table 3).

**Figure 1:**
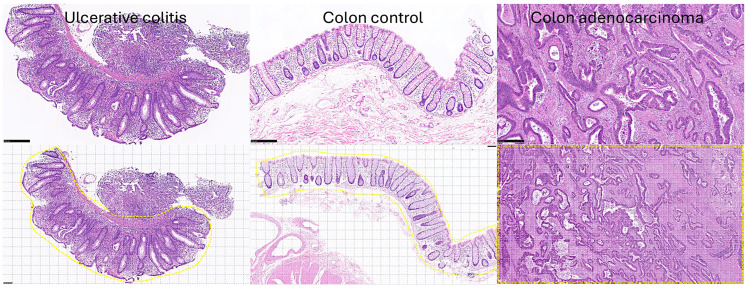
Histological types. Upper section: the three types of samples that were included in this study were ulcerative colitis (**left**), colon control (**middle**), and colorectal cancer (adenocarcinoma, **right**); original magnification 100×. Lower section: the whole-slide images were split into patches of 224 × 224 size (i.e., divided into multiple images of 224 px width and 224 px height). Only the diagnostic areas marked in yellow were used in the CNN analysis; original magnification 200×.

**Figure 2:**
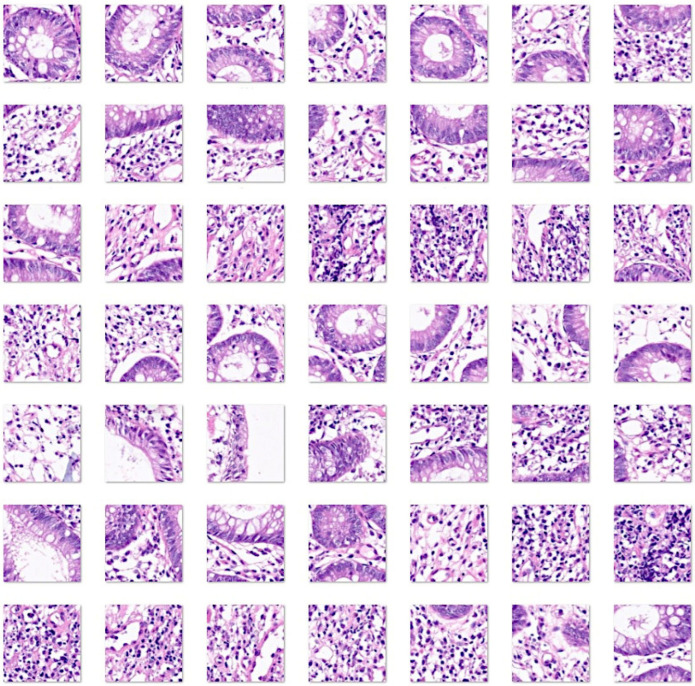
Images of ulcerative colitis. Whole-slide images at original magnification 200× were split into image patches of 224 × 224 size (i.e., divided into multiple images of 224 px width and 224 px height). Ulcerative colitis is an idiopathic chronic inflammation that affects the colon mucosa. This disorder characteristically affects the rectum and extends toward proximal sections of the colon in a continuous manner. Microscopically, there are signs of active chronic colitis when untreated. Chronicity includes distorted architecture of the crypts, such as atrophy, irregular spacing, shortening, and branching; inflammation of the lamina propria with basal lymphoplasmacytosis; and Panet cell metaplasia or hyperplasia. Disease activity is confirmed by neutrophil infiltration of the muscosa, cryptitis, crypt abscess, or ulceration. Typically, inflammation is limited to the mucosa and submucosa, and granulomas and fissuring ulcers are absent. Well-established disease can be associated with dysplasia of the epithelium, either low-grade or high-grade. The most commonly used score to evaluate the histological features is the Geboes score [93,94,95,96,97].

**Figure 3:**
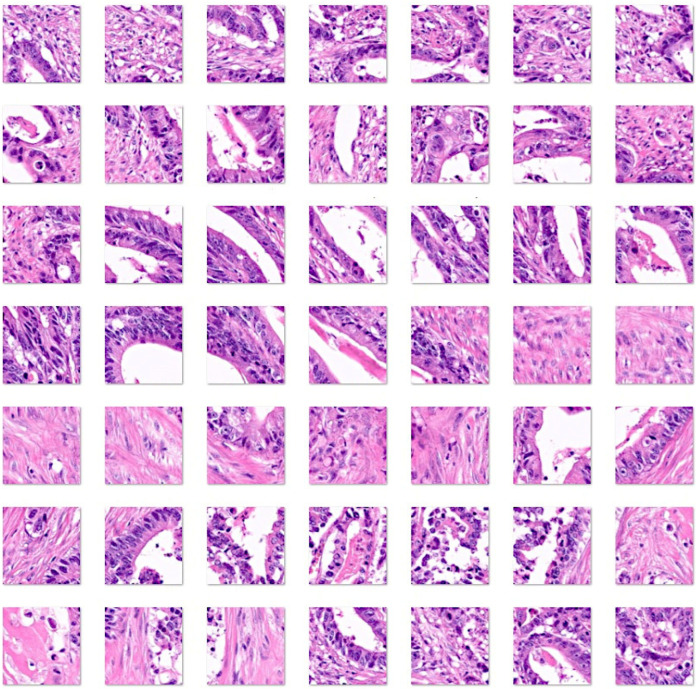
Images of colorectal cancer (adenocarcinoma). Original magnification 200×. Whole-slide images were split into patches of 224 × 224 size. Adenocarcinoma of the colon is a glandular neoplasm that accounts for approximately 98% of all colonic cancers. Patients with inflammatory bowel disease, polyposis, and Lynch syndrome [98,99] are at a higher risk of developing colorectal cancer. Most cases display high or moderate differentiation of the carcinoma glands accompanied by marked growth of the fibrous connective tissue, known as desmoplasia [100,101]. The glands can show a cribriform pattern and are filled with necrotic debris. Adenocarcinomas are characterized by epithelial cells with stretched and stratified nuclei, which create complex glandular structures. The nuclei exhibit polymorphism and loss of polarity. The tumor immune microenvironment exhibits variable infiltration of inflammatory cells. There are several recognized subtypes, including adenoma-like, adenosquamous, mucinous, micropapillary, signet-ring, serrated, and sarcomatoid [93,101,102,103,104,105,106,107].

**Figure 4:**
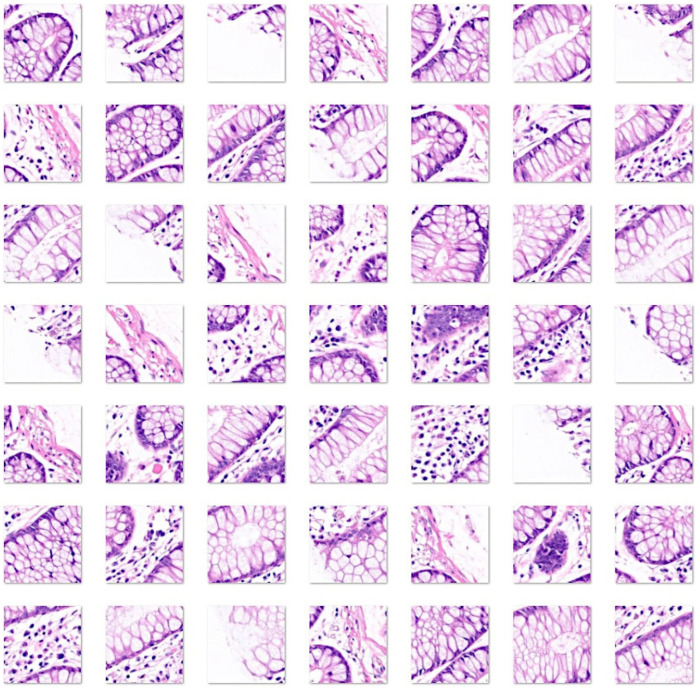
Images of the colon control. Original magnification 200×. Whole-slide images were split into patches of 224 × 224 size. The colonic mucosa functions primarily to absorb water and electrolytes, a process carried out by absorptive columnar cells, and to produce mucus for lubrication, which is secreted by goblet cells. The mucosa comprises the epithelium, lamina propria, and muscularis mucosa. The epithelium invaginates and forms glands (crypts), where at its base there is also the presence of enteroendocrine, Paneth cells, and stem cells. The lamina propria is rich in capillaries and lymphatics. Loose connective tissue and nerve plexuses are found in the submucosa. The muscularis propria has an inner circular layer and an outer longitudinal layer, and within them, the Auerbach nerve plexus is located. The outer layers are the subserosa and serosa [93,108,109].

Figure 1, Figure 2, Figure 3 and Figure 4 show characteristic H&E images of the different diagnoses and the splitting of images.

## 3. Results

### 3.1. Clinicopathological Characteristics and Conventional Histological Analysis

**Figure 5:**
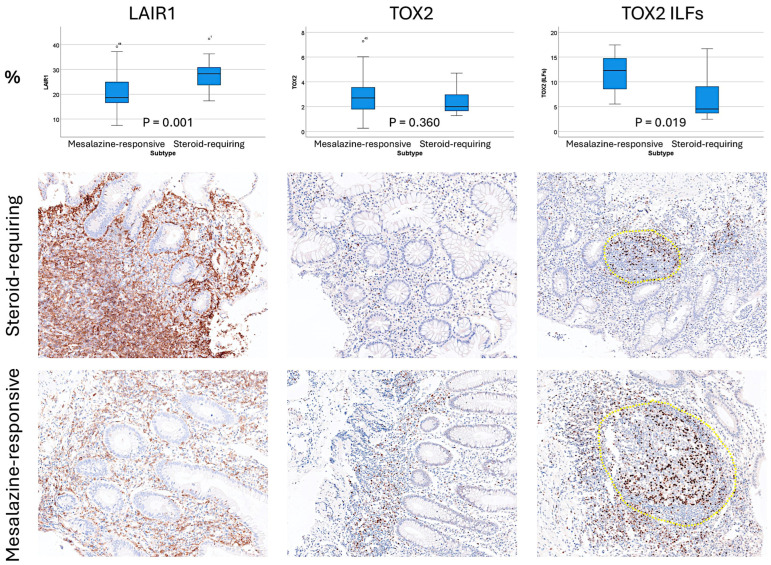
Conventional immunohistochemical analysis of LAIR1 and TOX2 in ulcerative colitis. LAIR1 and TOX2 are two new immuno-oncology markers that target cells of the microenvironment. TOX2 is comparable to PD-1. In comparison to mesalazine-responsive ulcerative colitis, the steroid-requiring type was characterized by higher protein expression of LAIR1 (20.74% ± 7.48 vs. 28.18% ± 6.26, *p* = 0.001) and lower TOX2-positive cells in the isolated lymphoid follicles (ILFs) (11.74% ± 3.47 vs. 7.03% ± 5.03, *p* = 0.019). ILFs, isolated lymphoid follicles of the lamina propria. Original magnification 200×. The isolated lymphoid follicles are highlighted using a yellow circle.

The clinicopathological characteristics of the series are shown in Table 4. The number of cases in the series was 35, with a mean age of 38.4 years and a male/female ratio of 20/35. Most of the biopsies were from the rectum, which was the most pathological area. Most cases had an endoscopic Baron score of 1 and 2. The most frequent histologic Geboes scores were 2 to 4. In comparison to mesalazine-responsive ulcerative colitis, the steroid-requiring type was characterized by higher protein expression of LAIR1 (20.74% ± 7.48 vs. 28.18% ± 6.26, *p* = 0.001) and lower TOX2-positive cells in the isolated lymphoid follicles (ILFs) (11.74% ± 3.47 vs. 7.03% ± 5.03, *p* = 0.019) (Table 4 and Figure 5).

### 3.2. Image Classification Based on Transfer Learning from ResNet-18

**Figure 6:**
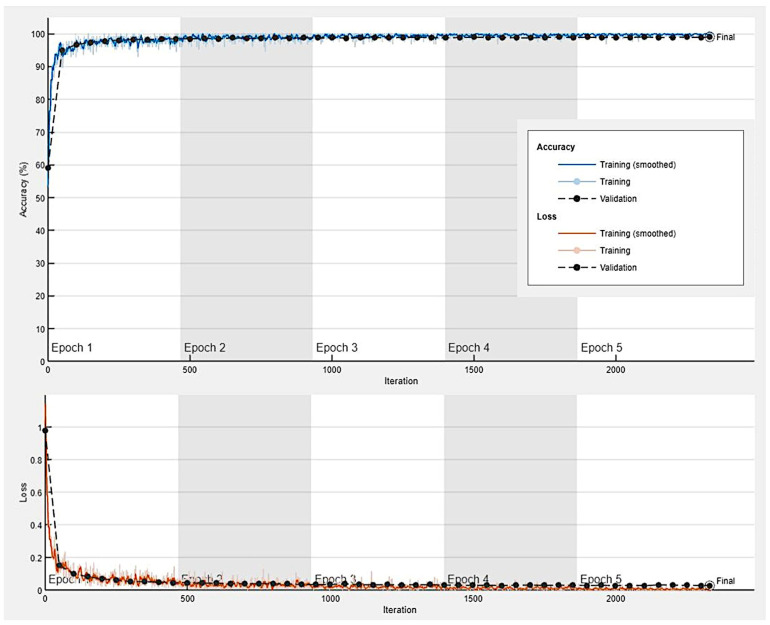
Network performance during training and validation. The data (image patches) were partitioned into a training set (70% of the images) to train the network and a validation set (10%) to test the performance of the network during the training; a test set (20%) was used as a holdout to test the performance of the trained network on new data. This figure shows the accuracy and loss during the training (70%) and validation (10%) sets. The CNN was based on transfer learning from ResNet-18.

Transfer learning using the pre-trained ResNet-18 CNN was used to classify the image patches of ulcerative colitis, colorectal cancer (adenocarcinoma), and colon control. The data (image patches) were partitioned into a training set (70% of the images) to train the network, a validation set (10%) to test the performance (accuracy and loss) of the network during training, and a test set (20%) as a holdout (new data) to test the performance on new data. The network performance during training and validation is shown in Figure 6. The network achieved high accuracy and low loss during the first 100 iterations.

**Figure 7:**
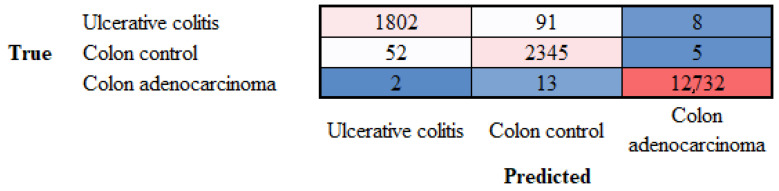
Confusion matrix of the test dataset (new data). The data were partitioned into a training set (70% of the image patches) to train the network and a validation set (10%) to test the performance of the network during the training; a test set (20%) was used as a holdout to test the performance of the trained network on new data.

After training, new images of the test set (holdout) were classified using the trained CNN. The result achieved a performance of 99% (accuracy). The confusion matrix is shown in Figure 7.

The classification performance for each diagnosis is presented in Table 5. Raw data is shown in Supplementary Material.

### 3.3. Grad-CAM Heatmap Analysis

**Figure 8:**
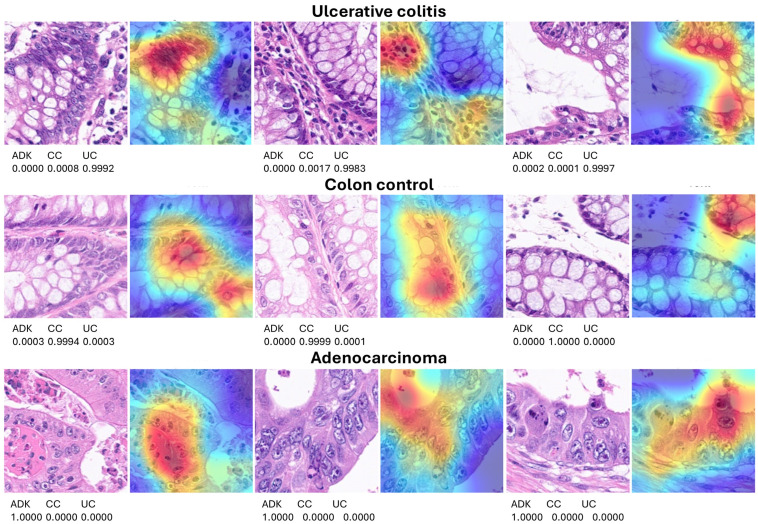
Explanation of network predictions using Grad-CAM. Grad-CAM was used to visualize which regions of the image were important for the classification decision (diagnosis) of the network. The most relevant regions are highlighted in red (jet colormap). The prediction scores for each diagnosis are shown below each hematoxylin and eosin (H&E) image. ADK, adenocarcinoma (colorectal cancer); CC, colon control; UC, ulcerative colitis. Original magnification 200× (whole-slide images were split into patches of 224 × 224 size).

The Grad-CAM heatmap was used to visualize which regions of the images were important to the classification decision of the network. This method uses the classification score gradient relative to the final convolutional feature map. The regions of the images with large values for the Grad-CAM maps had the greatest effect on the network score for that diagnosis (image classification). Some examples of correctly classified images are shown in Figure 8; the network focused on the epithelial layer and inflammatory components of the lamina propria.

**Figure 9:**
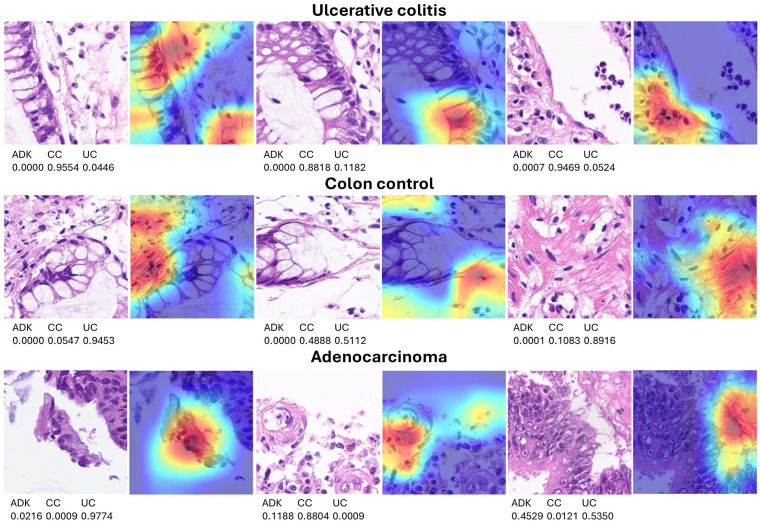
Grad-CAM analysis of incorrectly classified images. Grad-CAM analysis was used to visualize which regions of the image were important for the classification decision (diagnosis) of the network. In most cases, the classification errors occurred because the network focused on incorrect areas within the image or because the image itself was not diagnostic from a histopathological point of view. Original magnification 200× (whole-slide images were split into patches of 224 × 224 size).

In a few instances, the classification of images was incorrect, resulting in discrepancies between diagnosis and prediction. A review of these cases showed that, in most instances, the discrepancies arose because the images were not diagnostic from a histopathological point of view or because the network was focusing on an incorrect region of the image during classification, as shown in the Grad-CAM analysis (Figure 9).

### 3.4. Differentiation Between Steroid-Requiring and Mesalazine-Responsive Ulcerative Colitis

**Figure 10:**
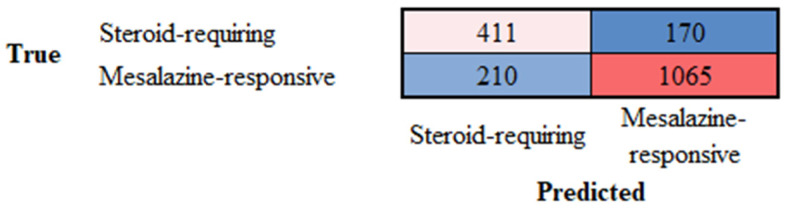
Confusion matrix of the test dataset for the classification of steroid-requiring and nonsteroid-requiring/mesalazine-responsive. The test dataset included new data (holdout, 20%). The analysis was based on transfer learning from ResNet-18. The accuracy was 79.53%.

Ulcerative colitis can be divided into two clinical groups based on the requirement for steroids to control colon inflammation. Transfer learning from ResNet-18 was performed to predict and classify the H&E images of ulcerative colitis into the two subtypes of steroid-requiring (“aggressive”) and mesalazine-responsive (“benign”). In the test set, the accuracy was 79.53%. The confusion matrix is shown in Figure 10, and the network performance is shown in Table 6.

### 3.5. Differentiation Between Steroid-Requiring (SR) and Mesalazine-Responsive Ulcerative Colitis Using LAIR1 Immunohistochemistry

**Figure 11:**
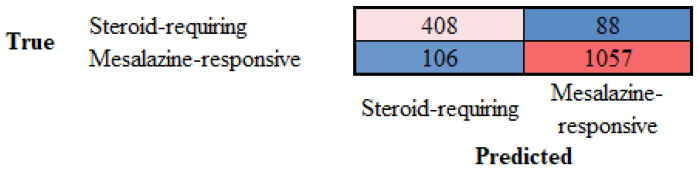
Confusion matrix of the test dataset for the classification of steroid-requiring and nonsteroid-requiring/mesalazine-responsive using LAIR1 immunohistochemistry. The test dataset included new data (holdout, 20%). The analysis was based on transfer learning from ResNet-18. The accuracy was 88.31%.

Ulcerative colitis can be divided into two clinical groups based on the requirement for steroids to control colon inflammation, as described in Section 3.3. The analysis of H&E highlighted the importance of both the epithelial and inflammatory components. LAIR1 is a new marker of the immune microenvironment and an immuno-oncology target. Therefore, transfer learning from ResNet-18 was performed to predict and classify the LAIR1 images of ulcerative colitis into the two subtypes of steroid-requiring (“aggressive”) and mesalazine-responsive (“benign”). In the test set, the accuracy was 88.31%. The confusion matrix is shown in Figure 11, and the network performance is shown in Table 7.

**Figure 12:**
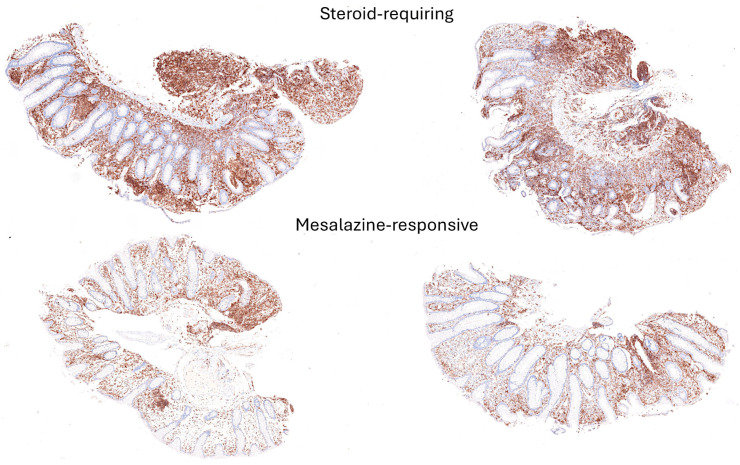
LAIR1 immunohistochemistry in the ulcerative colitis dataset and classification of steroid-requiring and nonsteroid-requiring/mesalazine-responsive ulcerative colitis cases. Overall, the inflammatory component was higher in the steroid-requiring cases. Original magnification 200×.

**Figure 13:**
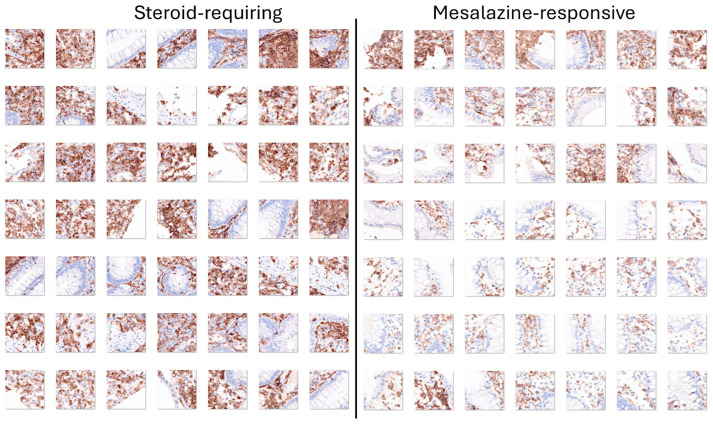
Examples of the split images of LAIR1 immunohistochemistry in the ulcerative colitis dataset. The split images were used as input data in the CNN, which managed to classify between steroid-requiring and nonsteroid-requiring/mesalazine-responsive ulcerative cases. Overall, the inflammatory component was higher in the steroid-requiring cases. Original magnification 200× (whole-slide images were split into patches of 224 × 224 size).

Examples of steroid-requiring and mesalazine-response ulcerative colitis and characteristic LAIR1 immunohistochemistry are shown in Figure 11. Evaluation of the whole-tissue images of LAIR1 showed that steroid-requiring patients had a more prominent inflammation of the lamina propria, as shown in Figure 12 and Figure 13.

### 3.6. Differentiation Between Steroid-Requiring (SR) and Mesalazine-Responsive Ulcerative Colitis Using TOX2 Immunohistochemistry

**Figure 14:**
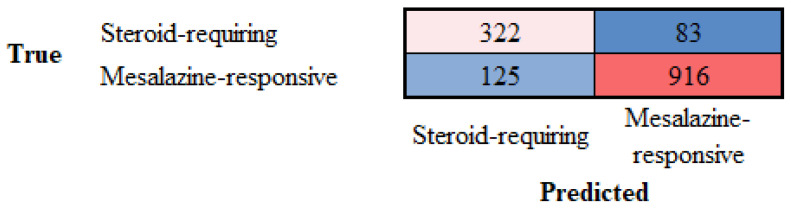
Confusion matrix of the test dataset for the classification of steroid-requiring and nonsteroid-requiring/mesalazine-responsive using TOX2 immunohistochemistry. The test dataset included new data (holdout, 20%). The analysis was based on transfer learning from ResNet-18. The accuracy was 85.62%.

TOX2 (TOX high mobility group box family member 2) is a new marker of the immune microenvironment and an immuno-oncology target. Transfer learning using ResNet-18 classified the TOX2 images of ulcerative colitis into steroid-requiring and mesalazine-responsive. In the test set, the accuracy was 85.62%. The confusion matrix is shown in Figure 14, and the network performance is shown in Table 8.

**Figure 15:**
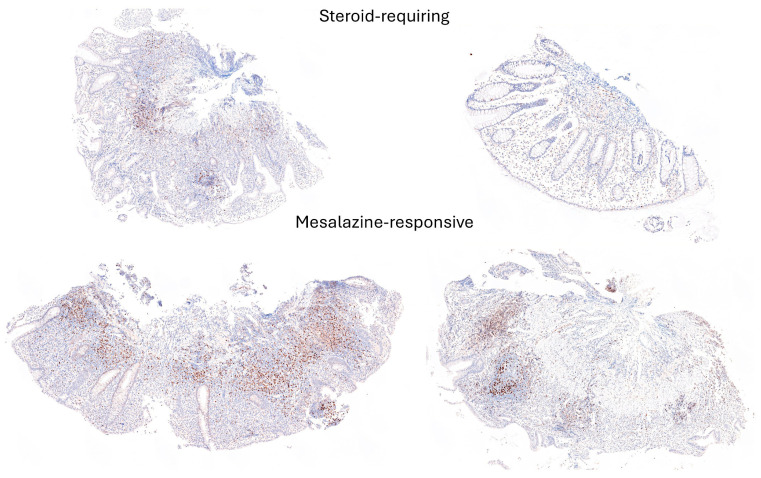
TOX2 immunohistochemistry in the ulcerative colitis dataset and classification of steroid-requiring and nonsteroid-requiring/mesalazine-responsive ulcerative colitis cases. Overall, the TOX2-positive inflammatory component was higher in the mesalazine-responsive cases. Original magnification 100×.

**Figure 16:**
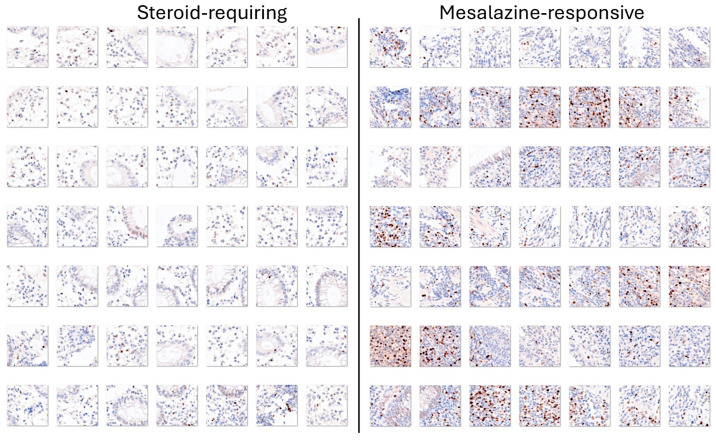
Examples of the split images of TOX2 immunohistochemistry in the ulcerative colitis dataset. The split images were used as input data in the CNN, which managed to classify between steroid-requiring and nonsteroid-requiring/mesalazine-responsive ulcerative cases. Overall, the TOX2-positive inflammatory component was higher in the mesalazine-responsive cases. Original magnification 200× (whole-slide images were split into patches of 224 × 224 size).

Examples of steroid-requiring and mesalazine-response ulcerative colitis and characteristic TOX2 immunohistochemistry are shown in Figure 14. Evaluation of the whole-tissue images of TOX2 showed that the TOX2-positive inflammatory component was higher in mesalazine-responsive cases, as shown in Figure 15 and Figure 16.

### 3.7. Image Classification Using Transfer Learning with Other Convolutional Neural Networks

The performance of ResNet-18 using H&E images was compared to other CNNs. Transfer learning using several pre-trained CNN was used to classify images of ulcerative colitis, colorectal cancer (adenocarcinoma), and colon control. The network performance of the validation test set (holdout, new data) is shown in Table 9 and Appendix A. ResNet-18 demonstrated strong performance with an accuracy of 99%. However, when all CNNs were run, the best results were obtained using DenseNet-201 (99.3%), ResNet-50 (99.14%), Inception-v3 (99.13%), and ResNet-101 (99.1%). Notably, the NasNet-Large CNN required a significantly longer training time (12,495 min 37 s) than the other models.

### 3.8. Image Classification of Additional Cases of Colorectal Cancer Using the ResNet-18 Trained Network

In this study, the image patches of the three diagnoses were pooled and later split into training, validation, and test sets. However, this strategy can create an information leak. Therefore, an additional independent series of 10 cases of colorectal cancer (adenocarcinoma) were classified to confirm the performance of the trained CNN (based on ResNet-18 architecture). In this analysis, each patient was analyzed independently. The CNN classified all cases as adenocarcinoma. The results are shown in Table 10.

### 3.9. Image Classification of Additional Cases of Mesalazine-Responsive Ulcerative Colitis with Absent/Mild Histological Changes Using the ResNet-18 Trained Network

An additional independent series of 10 cases of mesalazine-responsive ulcerative colitis with absent/mild histological epithelial changes was analyzed to confirm the performance of the trained CNN (based on ResNet-18 architecture). In this analysis, each patient was analyzed independently. The CNN classified most of the cases as colon control. Therefore, the CNN did not outperform the diagnostic abilities of the experienced medical pathologist, who also incorporated clinical variables into their final diagnosis. Notably, the Grad-CAM analysis showed that the CNN was focusing on the epithelial layer. The results are shown in Table 11.

## 4. Discussion

Ulcerative colitis is a chronic inflammatory bowel disease that primarily affects the mucosal layer of the colon recurrently. It usually affects the rectum and extends continuously toward the proximal segments of the colon [5,110]. Patients usually present with diarrhea that may include blood, with a gradual and progressive onset of symptoms [5,45,110]. Disease evaluation includes history, laboratory studies, endoscopy, and biopsy. Several factors affect the disease course, including age at diagnosis, mucosal healing, extension of colitis, and smoking. Chronic complications include stricture, dysplasia, and colorectal cancer [49].

The endoscopic findings are nonspecific and include loss of vascular marking, granular mucosa, petechiae, exudates, edema, erosions, friability, ulcerations, and bleeding [111]. Endoscopic biopsies show neutrophilic infiltration with cryptitis, crypt abscesses, and ulcerations when the disease is active. Chronic disease involves crypt architectural alterations, chronic inflammation of the lamina propria with lymphoplasmocytosis, and Paneth cell metaplasia or hyperplasia [93,94,108]. This study analyzed images of ulcerative colitis using a convolutional neural network (CNN).

A CNN is a deep learning technique in machine learning, trained using a large dataset of images. In this study, a CNN was used to analyze whole-slide images (WSI) from hematoxylin and eosin (H&E) microscope pathology slides of ulcerative colitis, colon control, and colorectal cancer (adenocarcinoma). Artificial intelligence (AI) refers to systems capable of simulating human intelligence by mimicking cognitive functions such as learning and problem solving. AI can be divided into two types: artificial general intelligence, or strong AI, which exhibits generalized human cognitive abilities [112,113,114]; and narrow AI, or weak AI, which focuses on specific tasks of human intelligence [115,116]. There are two types of narrow AI: rule-based AI follows predefined machine rules, while example-based AI learns patterns from provided examples [117]. This study employed narrow AI for histological analysis to classify gut images. The slides were digitalized using a slide scanner that converted glass slides into digital data. In this study, both endoscopic biopsy and surgical resection specimens were used. The CNN could differentiate the three types of diagnoses with high performance (99%). However, careful consideration is necessary in the differential diagnosis of ulcerative colitis.

The evaluation and establishment of a diagnosis of ulcerative colitis require the exclusion of other causes of colitis through a combination of patient history, laboratory studies, endoscopic imaging, and colon biopsies. In the history, other causes of colitis should be excluded, including parasitic infections, sexually transmitted infections (Neisseria gonorrhoeae and herpes simplex virus), atherosclerotic disease (chronic colonic ischemia), abdominal/pelvic radiation, and the use of non-steroidal anti-inflammatory drugs. The stool should also be tested for C. trachomatis, N. gonorrhoeae, HSV, and Treponema pallidum. The endoscopic and histological findings of ulcerative colitis are not specific. However, differentiating ulcerative colitis from Crohn’s disease is important because of its different prognoses and treatments [118]. A comprehensive review and update on ulcerative colitis, including an extended differential diagnosis, was described by Gajendran et al. [119]. Among the relevant diseases, neoplasm, Crohn’s disease, and Celiac disease were highlighted [119]. In this study, the image classification comprised cases of ulcerative colitis and colorectal cancer (adenocarcinoma). We recently performed histological image classification of Celiac disease, small intestine control, unspecific duodenal inflammation, and Crohn’s disease [71]. Narrow artificial intelligence (AI) is designed to perform tasks that typically require human intelligence; however, it operates within limited constraints and is task-specific [71]. In future, integrated analysis can be performed.

Computer vision provides a series of algorithms for visual inspection, object detection, and tracking, as well as feature detection, extraction, and matching. Pre-trained object detection can be performed using the YOLO, SSD, and ACF algorithms. Semantic and instance segmentation methods include U-Net, SOLO, and the Mask R-CNN [120]. Image classification can be performed using vision transformers such as ViT. In this study, we employed pre-trained image classification neural networks. These pre-trained networks had already learned to extract the characteristics of natural images. Therefore, we used them as a starting point to learn the new task of classifying ulcerative colitis, colonic control, and colorectal cancer (adenocarcinoma). Several CNNs were used, with the highest accuracies achieved by DenseNet-201 (99.30% accuracy), ResNet-50 (99.14%), Inception-v3 (99.13%), ResNet-101 (99.1%), and ResNet-18 (99%). The most relevant features of a CNN are accuracy, speed, and size. In this study, ResNet-18 was the most convenient CNN to use due to its accuracy and relative prediction time using GPU. NASNet-Large also delivered high accuracy; however, despite its high accuracy, its prediction time was the slowest.

Explainable AI (XAI) allows humans to understand and trust results by providing clear and understandable explanations [121,122,123,124]. In computer vision, a common method to demonstrate XAI techniques is to overlay an explanation on the image using an explanation heatmap. In this study, Grad-CAM was used [125,126]. The other XAI methods are LIME and SHAP [127]. Grad-CAM uses a gradient-weighted class activation mapping technique to understand how a deep learning network makes its classification decisions. As shown in the results section, the Grad-CAM analysis confirmed that the CNN focused on the correct area of the images to perform classification. Interestingly, in the discordant cases, the Grad-CAM analysis revealed incorrect focus by the CNN. In addition, some discordant cases were due to nondiagnostic images.

In the last 5 years there have been several reports on ulcerative colitis and deep learning. Most applications have focused on endoscopic image analysis [128,129,130,131,132,133,134,135]. However, some research has been published regarding histological images. Rymarczk et al. used deep learning models to analyze the histological disease activity in Crohn’s disease and ulcerative colitis [136]. Vande Casteele et al. used a deep learning algorithm to identify eosinophils in colonic biopsies of active ulcerative colitis [137]. Ohara et al. applied deep learning to detect goblet cell mucus [138]. Peyrin-Biroulet used an AI algorithm to measure the Nancy index in histological images of patients with ulcerative colitis [139]. Our study differs from previous research in that it not only diagnosed ulcerative colitis in comparison to colon controls but also identified images of colorectal cancer. To the best of our knowledge, no similar study has been conducted to date.

LAIR1 protein functions as an inhibitory receptor that is expressed in monocytes, natural killer cells, and T and B lymphocytes. LAIR1 is also expressed in macrophages, where it regulates their activation [140]. In lymphoma, LAIR1 expression by tumor-associated macrophages has been described [141]. In ulcerative colitis, the expression of LAIR1 has been recently described by Hassan-Zahraee et al. [142]. However, to the best of our knowledge, LAIR1 has not been described in other inflammatory bowel diseases. In our study, LAIR1 was expressed by cells of the immune microenvironment, and the CNN managed to classify between steroid-requiring (SR) and nonsteroid requiring (non-SR) ulcerative colitis. Therefore, LAIR1 is a promising immuno-oncology marker in ulcerative colitis.

TOX2 (TOX high mobility group box family member 2) is a transcription factor related to T cell exhaustion [143], which is a broad term used to describe the T cell functions in conditions of chronic antigen stimulation, such as inflammatory bowel disease and response to tumors [144]. Evidence indicates that TOX2 is expressed in T follicular helper (TFH) cells, similar to the PD-1 marker, and may suppress CD4 cytotoxic T cell differentiation [145]. TOX2 has been related to the survival of patients with acute myeloid leukemia [144] and the pathogenesis of atopic dermatitis [146]. Due to its relationship with PD-1, TOX2 is a promising immuno-oncology marker in ulcerative colitis as well.

This study has the limitation of the number of samples used for the CNN analysis. Therefore, in future research, the series should be increased and the trained network retrained. There is also a limitation regarding the classification of mesalazine-responsive ulcerative colitis with absent/mild histological changes.

Notably, this study highlighted clinical implications. Conventional clinicopathological analysis showed that steroid-requiring ulcerative colitis was characterized by higher endoscopic Baron and histologic Geboes scores, as well as increased LAIR1 expression in the lamina propria, but lower TOX2 expression in isolated lymphoid follicles (all *p* values < 0.05) compared to mesalazine-responsive ulcerative colitis. The use of CNNs holds promise, but validation will require larger series of cases, particularly in mesalazine-responsive cases.

## 5. Conclusions

A convolutional neural network demonstrates strong performance in predicting ulcerative colitis, colon control, and colorectal cancer (adenocarcinoma). LAIR1 and TOX2 have emerged as two promising immuno-oncology markers for ulcerative colitis.

## Figures and Tables

**Table 1:** Pathogenesis of inflammatory bowel disease.

Mechanisms	Key Players
Dysregulation of the epithelial barrier	Alterations of the mucus, increased number of bacteria within the mucus, and increased intestinal permeability [6,7,8].
Dysregulation of immune cells	Increased recruitment and activation of immune cell, including myeloid inflammatory cells, natural killer cells, T cells, B cells, plasma cells, neutrophils, and other leukocytes [9,10,11,12,13,14,15,16,17].
Dysregulation of immune regulators and inflammatory cytokines	CD4 + T lymphocytes, interferon (IFN)-gamma, Th1, Th2, Th17, FOXP3 + regulatory T lymphocytes (Tregs), IL-10, TGFB, CD8 + cytotoxic T lymphocytes [18,19,20,21,22,23,24,25].
Microbes	Alterations in the diversity and density of bacteria [26,27,28,29], specific microbial components, intestinal viruses [9,30,31,32], and fungi [33,34,35].
Genetic susceptibility	Over 240 different susceptibility loci, *NOD2*, *ATG16L1*, *NADPH*, and immune-related (Th17/IL-23, IL-10, TNFSF15, cytokine, adaptive immune response, and epithelial pathways) [14,36,37,38,39,40,41,42,43,44].

**Table 2:** Design and training parameters of the convolutional neural network.

ResNet-18-Based CNN	Training (70%)	Validation (10%)	Training Options
Input type: image patches Output type: classification Number of layers: 71 Number of connections: 78	Observations: 59,677 Classes: 3 Ulcerative colitis: 6497 Colorectal cancer: 44,608 Colon control: 8572	Observations: 8525 Classes: 3 Ulcerative colitis: 928 Colorectal cancer: 6372 Colon control: 1225	Solver: sgdm Initial learning rate: 0.001 MiniBatch size: 128 MaxEpochs: 5 Validation frequency: 50 Iterations: 2330 Iterations per epoch: 466

Based on transfer learning of ResNet-18. Convolutional neural network, CNN.

**Table 3:** Transfer learning using pre-trained neural networks.

Name [References]	Model Name Argument	Depth	Size (MB)	Parameters (Millions)	Image Input Size
AlexNet [81]	“alexnet”	8	227	61	227-by-227
DenseNet-201 [82]	“densenet201”	201	77	20	224-by-224
EfficientNet-b0 [83]	“efficientnetb0”	82	20	5.3	224-by-224
GoogLeNet [84,85]	“googlenet”	22	27	7	224-by-224
	“googlenet-places365”				
Inception-v3 [86]	“inceptionv3”	48	89	23.9	299-by-299
MobileNet-v2 [87]	“mobilenetv2”	53	13	3.5	224-by-224
NASNet-Large [88]	“nasnetlarge”	*	332	88.9	331-by-331
NASNet-Mobile [88]	“nasnetmobile”	*	20	5.3	224-by-224
ResNet-18 [89]	“resnet18”	18	44	11.7	224-by-224
ResNet-50 [89]	“resnet50”	50	96	25.6	224-by-224
ResNet-101 [89]	“resnet101”	101	167	44.6	224-by-224
ShuffleNet [90]	“shufflenet”	50	5.4	1.4	224-by-224
VGG-16 [91]	“vgg16”	16	515	138	224-by-224
VGG-19 [91]	“vgg19”	19	535	144	224-by-224
Xception [92]	“xception”	71	85	22.9	299-by-299

* The NASNet-Mobile and NASNet-Large neural networks do not consist of a linear sequence of modules.

**Table 4:** Clinicopathological characteristics of ulcerative colitis.

Variable No. (%)	Mesalazine -Responsive	Steroid -Requiring	All Cases	*p* Value
Number of patients	22	13	35	
Age (mean ± STD)	43.7 ± 13.6	29.5 ± 17.6	38.4 ± 16.4	0.012
Sex (male/female)	14/22 (63.6)	6/13 (46.2)	20/35 (57.1)	0.481
Colon biopsy location				
Ascending	0/22 (0)	1/13 (7.7)	1/35 (2.9)	0.009
Transverse	0/22 (0)	2/13 (15.4)	2/35 (5.7)	
Descending	2/22 (9.1)	3/13 (23.1)	5/35 (14.3)	
Sigmoid	2/22 (9.1)	3/13 (23.1)	5/35 (14.3)	
Rectum	18/22 (81.8)	4/13 (30.8)	22/35 (62.9)	
Endoscopic Baron score				
1	13/22 (59.1)	2/13 (15.4	15/35 (42.9)	0.009
2	9/22 (40.9)	8/13 (61.5)	17/35 (48.6)	
3	0/22 (0)	3/13 (23.1)	3/35 (8.6)	
Histologic Geboes score				
1	2/22 (9.1)	0/13 (0)	2/35 (5.)	0.101 (0.007 *^1^)
2	13/22 (59.1)	4/13 (30.8)	17/35 (48.6)	
3	5/22 (22.7)	3/13 (23.1)	8/35 (22.9)	
4	2/22 (9.1)	5/13 (38.5)	7/35 (20)	
5	0/22 (0)	1/13 (7.7)	1/35 (2.9)	
Immuno-oncology markers				
LAIR1	20.74% ± 7.48	28.18% ± 6.26	23.28% ± 7.86	0.001
TOX2	2.98% ± 1.78	2.39% ± 1.05	2.79% ± 1.59	0.360
TOX2 isolated lymphoid follicles *^2^	11.74% ± 3.47	7.03% ± 5.03	10.37% ± 2.45	0.019

*^1^ Linear-by-linear association within the chi-square test. *^2^ Isolated lymphoid follicles were only present in 24 cases.

**Table 5:** Parameters of the network performance of the test dataset (holdout, new data).

Predicted Variable	Accuracy (%)	Precision (%)	Recall (%)	F1-Score (%)	Specificity (%)	False Positive Rate (%)
Ulcerative colitis	99.10	97.09	94.79	95.93	99.64	0.36
Adenocarcinoma	99.84	99.90	99.88	99.89	99.70	0.30
Colon control	99.06	95.75	97.63	96.68	99.29	0.71

Based on ResNet-18 transfer learning. Recall also refers to sensitivity and the true positive rate (TPR). False positive rate (FPR).

**Table 6:** Parameters of the network performance of the test dataset (holdout, new data) using H&E images.

Predicted Variable	Accuracy (%)	Precision (%)	Recall (%)	F1-Score (%)	Specificity (%)	False Positive Rate (%)
Steroid-requiring	79.53	66.18	70.74	68.39	83.53	16.47
Mesalazine-responsive	79.53	86.23	83.53	84.86	70.74	29.26

Based on ResNet-18 transfer learning. Recall also refers to sensitivity and the true positive rate (TPR). False positive rate (FPR).

**Table 7:** Parameters of network performance of the test dataset (holdout, new data) using LAIR1 immunohistochemistry.

Predicted Variable	Accuracy (%)	Precision (%)	Recall (%)	F1-Score (%)	Specificity (%)	False Positive Rate (%)
Steroid-requiring	88.31	79.38	82.26	80.79	90.89	9.11
Mesalazine-responsive	88.31	92.31	90.89	91.59	82.26	17.74

Based on ResNet-18 transfer learning. Recall also refers to sensitivity and the true positive rate (TPR). False positive rate (FPR).

**Table 8:** Parameters of the network performance of the test dataset (holdout, new data) using TOX2 immunohistochemistry.

Predicted Variable	Accuracy (%)	Precision (%)	Recall (%)	F1-Score (%)	Specificity (%)	False Positive Rate (%)
Steroid-requiring	85.62	72.04	79.51	75.59	87.99	12.01
Mesalazine-responsive	85.62	91.69	87.99	89.80	79.51	20.49

Based on ResNet-18 transfer learning. Recall also refers to sensitivity and the true positive rate (TPR). False positive rate (FPR).

**Table 9:** Performance comparison of CNN networks on the test set (holdout, new data) using H&E images.

Model	Accuracy (%)	Training Time
DenseNet-201	99.30	302 min 29 s
ResNet-50	99.14	24 min 30 s
Inception-v3	99.13	138 min 28 s
ResNet-101	99.10	252 min 45 s
ResNet-18	99.00	38 min 31 s
ShuffleNet	98.94	10 min 13 s
MobileNet-v2	98.89	22 min 27 s
NasNet-Large	98.88	12,495 min 37 s
GoogLeNet-Places365	98.86	17 min 16 s
VGG-19	98.80	439 min 48 s
EfficientNet-b0	98.79	55 min 25 s
AlexNet	98.77	8 min 23 s
Xception	98.66	497 min 23 s
VGG-16	98.65	365 min 54 s
GoogLeNet	98.60	17 min 5 s
NasNet-Mobile	98.58	88 min 34 s

This analysis was performed using transfer learning from several types of pre-trained convolutional neural networks (CNN) for image classification. Transfer learning using several pre-trained CNNs was used to classify images of ulcerative colitis, colorectal cancer (adenocarcinoma), and colon control.

**Table 10:** Image classification of 10 additional cases of colorectal adenocarcinoma using the trained network.

Classification	Case 1	Case 2	Case 3	Case 4	Case 5	Case 6	Case 7	Case 8	Case 9	Case 10
True	CRC	CRC	CRC	CRC	CRC	CRC	CRC	CRC	CRC	CRC
Predicted										
Adenocarcinoma	460	970	769	961	356	67	376	504	677	210
(%)	(98.08)	(99.18)	(96.13)	(100)	(83.76)	(82.72)	(96.66)	(98.82)	(96.58)	(50.60)
Ulcerative colitis	9	1	19	0	66	11	10	6	24	195
(%)	(1.92)	(0.10)	(2.38)	(0.00)	(15.53)	(13.58)	(2.57)	(1.18)	(3.42)	(46.99)
Colon control	0	7	12	0	3	3	3	0	0	10
(%)	(0.00)	(0.72)	(1.50)	(0.00)	(0.71)	(3.70)	(0.77)	(0.00)	(0.00)	(2.41)
Total image patches	469	978	800	961	425	81	389	510	701	415
(%)	(100)	(100)	(100)	(100)	(100)	(100)	(100)	(100)	(100)	(100)

In this analysis, each whole-slide image of each patient with colon adenocarcinoma was analyzed independently, and the image patches were analyzed using the previously trained ResNet-18 CNN. Colorectal cancer (CRC, adenocarcinoma).

**Table 11:** Image classification of 10 additional cases of mesalazine-responsive ulcerative colitis with absent/mild histological changes and an almost normal epithelial layer.

Classification	Case 1	Case 2	Case 3	Case 4	Case 5	Case 6	Case 7	Case 8	Case 9	Case 10
True	UC	UC	UC	UC	UC	UC	UC	UC	UC	UC
Baron score	1	1	1	1	2	2	2	2	2	1
Geboes score	2	4	2	2	2	1	3	2	4	1
Predicted										
Adenocarcinoma	0	81	438	1	8	5	2	1	11	0
(%)	(0.00)	(17.31)	(78.21)	1(0.28)	(1.29)	(1.69)	(0.56)	(0.31)	(2.64)	(0.00)
Ulcerative colitis	1	4	4	70	302	0	79	18	223	91
(%)	(0.46)	(0.85)	(0.71)	(19.83)	(48.79)	(0.00)	22.07	5.66	53.48	56.88
Colon control	217	383	118	282	309	291	277	299	183	69
(%)	99.54	81.84	21.07	79.89	49.92	98.31	77.37	94.03	43.88	43.13
Total image patches	218	468	560	353	619	296	358	318	417	160
(%)	(100)	(100)	(100)	(100)	(100)	(100)	(100)	(100)	(100)	(100)

In this analysis, each whole-slide image from each patient was analyzed independently, and the image patches were analyzed using the previously trained ResNet-18 CNN through transfer learning. These cases were characterized by absent or mild architectural changes in the epithelium, mild inflammation, and variable inflammation in some cases. The trained ResNet-18 CNN failed to properly classify these cases. Therefore, the CNN did not outperform the diagnostic abilities of the medical specialist in pathology, who also incorporated clinical variables into their final diagnosis.

## Data Availability

All the data, including methodology, are available upon request to Joaquim Carreras (joaquim.carreras@tokai.ac.jp). Additional data are located at Zenodo CERN and OpenAIRE Open Science repository https://doi.org/10.5281/zenodo.14287219.

## Supplementary Materials

The following supporting information can be downloaded at: https://www.mdpi.com/article/10.3390/cancers16244230/s1, Training and testing of neural networks (Text file).

## Appendix A. Confusion Matrices (Test Set, New Data) (Accuracy)

DenseNet-201 (99.3%)

Adenocarcinoma	12,741	3	0
Colon control	1	2362	29
Ulcerative colitis	3	84	1827
	Adenocarcinoma	Colon control	Ulcerative colitis

ResNet-50 (99.14%)

Adenocarcinoma	12,740	12	4
Colon control	3	2355	44
Ulcerative colitis	2	82	1808
	Adenocarcinoma	Colon control	Ulcerative colitis

Inception-v3 (99.13%)

Adenocarcinoma	12,737	12	5
Colon control	3	2350	37
Ulcerative colitis	5	87	1814
	Adenocarcinoma	Colon control	Ulcerative colitis

ResNet-101 (99.1%)

Adenocarcinoma	12,740	11	8
Colon control	0	2359	51
Ulcerative colitis	5	79	1797
	Adenocarcinoma	Colon control	Ulcerative colitis

ResNet-18 (99%)

Adenocarcinoma	12,732	13	2
Colon control	5	2345	52
Ulcerative colitis	8	91	1802
	Adenocarcinoma	Colon control	Ulcerative colitis

ShuffleNet (98.94%)

Adenocarcinoma	12,734	14	8
Colon control	7	2336	49
Ulcerative colitis	4	99	1799
	Adenocarcinoma	Colon control	Ulcerative colitis

MobileNet-v2 (98.89%)

Adenocarcinoma	12,734	19	24
Colon control	7	2334	40
Ulcerative colitis	4	96	1792
	Adenocarcinoma	Colon control	Ulcerative colitis

NasNet-Large (98.88%)

Adenocarcinoma	12,732	23	8
Colon control	5	2287	8
Ulcerative colitis	8	139	1840
	Adenocarcinoma	Colon control	Ulcerative colitis

GoogLeNet-Places365 (98.86%)

Adenocarcinoma	12,702	4	0
Colon control	24	2332	35
Ulcerative colitis	19	113	1821
	Adenocarcinoma	Colon control	Ulcerative colitis

VGG-19 (98.8%)

Adenocarcinoma	1270	19	13
Colon control	3	2371	109
Ulcerative colitis	2	59	1734
	Adenocarcinoma	Colon control	Ulcerative colitis

EfficientNet-b0 (98.79%)

Adenocarcinoma	12,733	8	8
Colon control	4	2311	49
Ulcerative colitis	8	130	1799
	Adenocarcinoma	Colon control	Ulcerative colitis

AlexNet (98.77%)

Adenocarcinoma	12,740	18	12
Colon control	2	2302	46
Ulcerative colitis	3	129	1798
	Adenocarcinoma	Colon control	Ulcerative colitis

Xception (98.66%)

Adenocarcinoma	12,718	30	12
Colon control	16	2302	43
Ulcerative colitis	11	117	1801
	Adenocarcinoma	Colon control	Ulcerative colitis

VGG-16 (98.65%)

Adenocarcinoma	12,744	26	59
Colon control	1	2363	85
Ulcerative colitis	0	60	1712
	Adenocarcinoma	Colon control	Ulcerative colitis

GoogLeNet (98.6%)

Adenocarcinoma	12,726	23	9
Colon control	5	2256	17
Ulcerative colitis	14	170	1830
	Adenocarcinoma	Colon control	Ulcerative colitis

NasNet-Mobile (98.58%)

Adenocarcinoma	12,724	18	4
Colon control	17	2320	88
Ulcerative colitis	4	111	1764
	Adenocarcinoma	Colon control	Ulcerative colitis
